# Effective suppression of efficiency droop in GaN-based light-emitting diodes: role of significant reduction of carrier density and built-in field

**DOI:** 10.1038/srep34586

**Published:** 2016-10-19

**Authors:** Yang-Seok Yoo, Jong-Ho Na, Sung Jin Son, Yong-Hoon Cho

**Affiliations:** 1 Department of Physics, Korea Advanced Institute of Science and Technology (KAIST), 291 Daehak-ro, Yuseong-gu, Daejeon 34141, Republic of Korea; 2 LG Innotek, LED Division, LED R&D Center, Paju 10842, Republic of Korea

## Abstract

A critical issue in GaN-based high power light-emitting diodes (LEDs) is how to suppress the efficiency droop problem occurred at high current injection while improving overall quantum efficiency, especially in conventional *c*-plane InGaN/GaN quantum well (QW), without using complicated bandgap engineering or unconventional materials and structures. Although increasing thickness of each QW may decrease carrier density in QWs, formation of additional strain and defects as well as increased built-in field effect due to enlarged QW thickness are unavoidable. Here, we propose a facile and effective method for not only reducing efficiency droop but also improving quantum efficiency by utilizing *c*-plane InGaN/GaN QWs having thinner barriers and increased QW number while keeping the same single well thickness and total active layer thickness. As the barrier thickness decreases and the QW number increases, both internal electric field and carrier density within QWs are simultaneously reduced without degradation of material quality. Furthermore, we found overall improved efficiency and reduced efficiency droop, which was attributed to the decrease of the built-in field and to less influence by non-radiative recombination processes at high carrier density. This simple and effective approach can be extended further for high power ultraviolet, green, and red LEDs.

GaN-based high power light-emitting diodes (LEDs) exhibit unsatisfactory characteristics; that is, the efficiency monotonically decreases with increasing injection current density, which is known as “efficiency droop”^1^. The origin of efficiency droop is still under debate, although many different contributions to efficiency droop have been discussed in the literature: carrier overflow due to the asymmetry of the electron-hole concentration or polarization^2^,^3^, Auger recombination^4^, junction heating effect^5^, carrier delocalization effect^6^, current crowding effect^7^, and density activated defect recombination (DADR) etc^8^. Recently, among them, research for efficiency droop in GaN-based LEDs has focused on reduction of non-radiative recombination processes at high carrier density region, such as Auger recombination and carrier overflow induced by strong internal electric field in the quantum well (QW).

Generally, a strong potential fluctuation induced by inhomogeneous In composition exists in InGaN-based QWs^9^. Carriers such as electrons and holes can be localized in the randomly distributed In-rich clusters that have sufficiently high localization energy even at room temperature. Since the radiative recombination of electrons and holes mainly occurs in the In-rich region, the effective active volume will be much smaller than the total active region^10^. Due to reduced effective active volume, actual carrier density inside the In-rich region should be much higher than that expected by uniform carrier distribution inside a QW. Large carrier density at the reduced effective active region contributes to a substantial increase of the Auger-like non-radiative recombination rate, which can lead to significant efficiency droop.

On the other hand, III-nitride semiconductors have a strong polarization along the *c*-direction due to their wurtzite crystal structure, and the polarization is determined by the chemical composition of the semiconductor as well as strain induced by distortion of the crystal lattice^11^. The strong polarization in active region may enhance leakage of electrons from multiple QWs (MQWs) to the *p*-GaN layer, and increased electron leakage to *p*-GaN layer prevents efficient hole injection into wells. Thus, the polarization-induced electric field in QWs is also attributed to efficiency degradation^12^.

Many efforts have been made to reduce the non-radiative recombination processes at high carrier density. Firstly, the polarization matching method of individual quantum barrier and well layers of the MQW active region by utilizing ternary InGaN or quaternary AlInGaN barriers was used for reducing the internal electric filed in InGaN QWs^13^,^14^. In addition, the engineering of band structure and nano or micro structures having semi- or non-polar facets were utilized to decrease the internal electric field^15^. Even though these techniques are efficient to diminish the internal electric field in the active region, the optimization of growth and fabrication conditions of these types of samples with other materials and structures than *c*-plane InGaN/GaN QWs become highly complicated, and many problems such as *p*-GaN growth and metal contact compared to the conventional LED structures still remain to be solved^16^. Secondly, the thick well, double heterostructure or simply increased QW number in active region were used to lower the effective carrier density in QWs^17^,^18^. Although these structures can reduce effective carrier density in larger active region, the material quality and recombination rate of QWs can be degraded due to the formation of additional detects and strain as well as increased quantum confined Stark effect caused by thicker active layer compared to conventional cases^19^. Therefore, it is essential to develop a practical and effective method to overcome the efficiency droop issue for conventional *c*-plane InGaN/GaN QWs without degrading material and optical qualities of active region.

Here, we propose an effective method for reducing the efficiency droop by using *c*-plane InGaN/GaN QWs structures having *thinner barriers* and *increased number of wells with fixed single well thickness* while keeping *the same total active layer region (i.e., thickness of whole wells and barriers)*. Using this structure, we found that the carrier density in the wells is reduced by augmented volume of total well region [i.e., (increased) well number × (fixed) well thickness] in the fixed total active region without degradation of material quality in QW. Moreover, the reduction of internal electric field in MQWs is observed, which comes from relaxation of compressive strain by utilizing thin barriers. By using this conventional *c*-plane InGaN-based LED structures, a dramatic reduction of internal electric field as well as carrier density is achieved without complicated band gap engineering and deterioration of material quality, improving overall internal quantum efficiency (IQE) and reducing the problem of efficiency droop.

## Results

**Figure 1:**
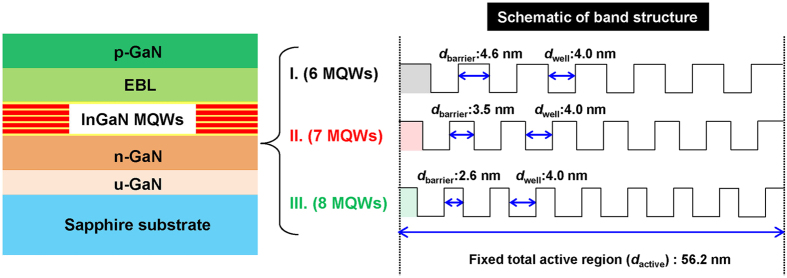
Schematic cross-section diagram of the epitaxial structure for samples I, II, and III, respectively. The *d*_active_ and *d*_well_ are fixed to 56.2 nm and 4 nm, respectively, while the *N*w increases with decreasing the *d*_barrier_.

The blue InGaN/GaN MQW LEDs used in this work were grown on *c*-plane sapphire substrates using a metal-organic chemical-vapor deposition (MOCVD) system. After depositing a low temperature GaN nucleation layer on the substrate, 5.3 μm thick *n*-type GaN:Si layer (*n*-doping = 3~7 × 10^18 ^cm^−3^) was grown followed by the growth of the active region. Then 60 nm thick *p*-type GaN:Mg (*p*-doping = 1 × 10^20 ^cm^−3^) was deposited upon the active region. A *p*-Al_0.2_Ga_0.8_N electron blocking layer having 20 nm thickness was used between the active region and the *p*-type GaN. The dimension of the fabricated LED chips was 1200 × 600 μm^2^. We prepared three kinds of LED samples, and the detailed structures of the samples are shown in Fig. 1. In order to keep the same material quality, the thicknesses of total active region (*d*_active_) and the single well thickness (*d*_well_) are fixed to 56.2 nm and 4 nm, respectively, which is the same in three LED samples, while the number of well (*N*_W_) increases with decreasing the barrier thickness (*d*_barrier_). The *d*_barrier_ decreases from 4.6 to 2.6 nm by adjusting growth time, and the *N*_W_ increases from 6 to 8 for samples I to III, keeping the same *d*_active_ and *d*_well_ for all the samples: *d*_barrier_ (*N*_W_) of 4.6 nm (6 MQWs), 3.5 nm (7 MQWs), and 2.6 nm (8 MQWs) with a fixed *d*_well_ of 4 nm, for samples I, II, and III, respectively.

**Figure 2:**
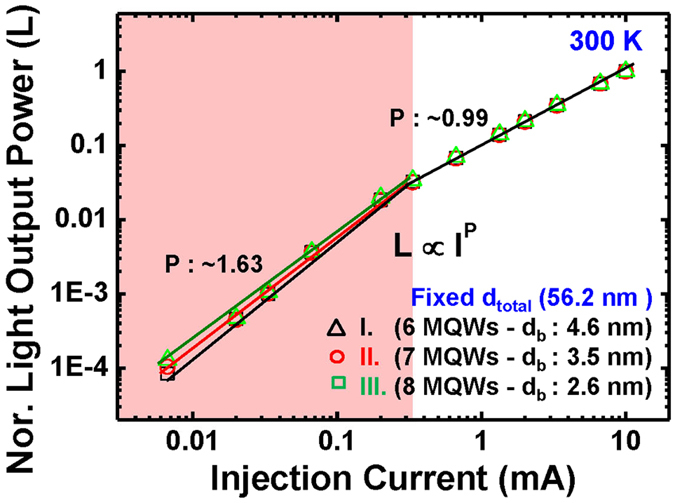
Electrical characteristic for samples I, II, and III. Semi-logarithmically plotted normalized light output power versus injection current at 300 K. The regions are divided two with variation of gradient. (red and white).

Figure 2 shows light output power (*L*) at the low injection current region for sample I, II, and III. Figure 2 shows the normalized *L* versus *I* plots for samples I to III. To avoid non-radiative recombination related to high injection current density such as Auger recombination (and/or carrier overflow), we conducted this experiment at low current injection region (below 10 mA). In red and white regions of Fig. 2, we observed the gradients were close to 2 and 1, respectively, indicating that the defect-related non-radiative recombination and the radiative recombination are dominant in the red region and the white region, respectively^20^. We note that from the change in their gradient, defect-related non-radiative recombination processes are not different between samples. From this result, we found that the degradation of material quality of the active region in samples was not cause by structural changes such as number of wells or barrier thickness.

**Figure 3:**
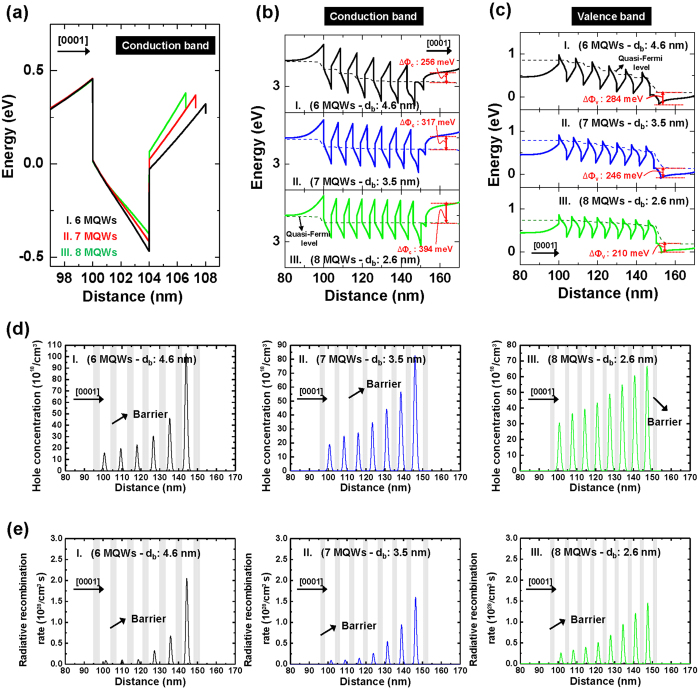
Analysis of carrier distribution by simulation for samples I, II, and III. (**a**) single conduction band diagram, (**b**) conduction band diagram (solid line) and quasi-Fermi level (dash line), (**c**) valence band diagram (solid line) and quasi-Fermi level (dash line), (**d**) hole concentration, and (**e**) radiative recombination rate for samples I to III at 50 A/cm^2^.

Generally, the non-uniform carrier distribution was observed by difference of mobility between electrons and holes, which was increased by strong internal electric field in QWs^21^. Consequently, the electrons and holes to the opposite within QWs are separated, which decreases the recombination process in QWs. In Fig. 3, the carrier distribution for samples I, II, and III was investigated through the simulation. The results of Fig. 3 were compared at 50 A/cm^2^. Figure 3a shows a simulated result of the conduction band edge (*E*_c_) of single QW for samples I to III. The growth direction is indicated by an arrow. The slope of *E*_c_ indicates the strength of the electric field in the well and the barrier. The sample with thinner *d*_barrier_ shows smaller slope (and hence smaller electric field) of *E*_c_ in the well than that of thicker *d*_barrier_. This result implies that the internal electric field in QWs is reduced as the barrier thickness decreases. Thus, we expect that decrease of kinetic energy for an electron with reducing the barrier thickness will increase the capture probability of electrons within QWs, and consequently decreases the carrier leakage. In addition, we investigated the effective conduction and valence band potential height of the AlGaN EBL close to the *p*-GaN. Figure 3b,c shows the conduction and valence band diagram (solid line) and quasi-Fermi levels (dash line), and the effective potential heights for electron escape in the conduction band (∆Φc) and that for hole injection in the valence band (∆Φv) of AlGaN EBL are investigated for samples I, II, and III. For the electrons, the effective conduction band height are 256 meV, 317 meV, and 394 meV, and for the holes, the effective valence band height are 284 meV, 246 meV, and 210 meV for samples I, II, and III, respectively. Unlike the electron, the hole distribution is non-uniform due to relatively large effective mass and low mobility of holes, and hole is commonly concentrated in QWs close to the *p*-GaN layer. In Fig. 3d,e, the hole concentration and radiative recombination rate are investigated. The gray lines as shown in Fig. 3d,e indicate the GaN barrier. The hole distribution of sample III became more uniform compare to the sample I and II. We thought that the hole injection was more efficient in sample with the thin barrier due to suppression of carrier leakage by decrease of internal electric field in QWs and the reduction of effective valence band height, resulting in increase of radiative recombination rate. The difference of carrier injection between samples was also observed from the result of turn-on-voltage. (see Supplementary Section I).

**Figure 4:**
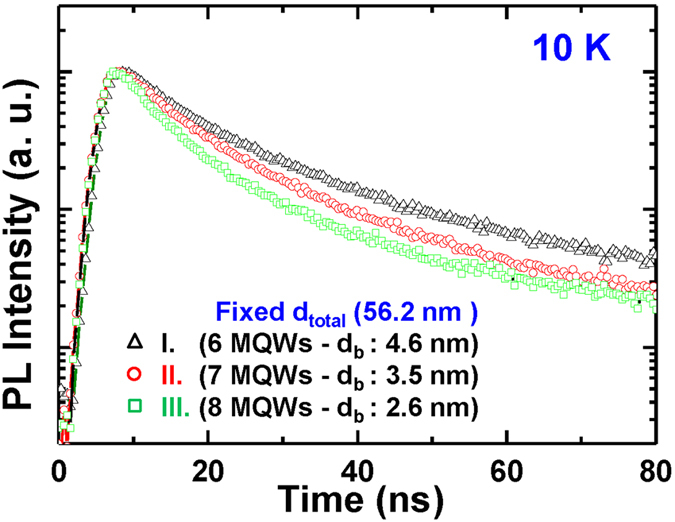
Time-resolved optical property for samples I, II, and III. Recombination lifetime from sample I to III measured at low temperature (10 K).

In order to observe any change of the internal electric field in QWs, measurement of photoluminescence (PL) decay time are carried out at low temperature (10 K) for the samples. The measured decay time is represented as follows: τ_mea_ = η_IQE_ × τ_rad_, where η_IQE_ and τ_rad_ are IQE and radiative recombination decay time, respectively. Generally, the IQE is assumed to be unity at sufficiently low temperature^22^. Thus, in this work, the decay time measured at low temperature is considered as the radiative recombination decay time. In Fig. 4, the lifetimes are shortened as *d*_barrier_ is reduced, which is attributed to an enhancement of the electron and the hole wave function overlap due to the decreased internal electric field in QWs with thinner *d*_barrier_^23^. In addition, the internal electric field between samples is investigated from difference of peak shift between samples. We observed that the peak wavelength was shorten, and difference of peak shift with injection current was reduced as the barrier thickness was decreased. We thought that these results were attributed to reduction of internal electric field in QWs. (see Supplementary Section II).

**Figure 5:**
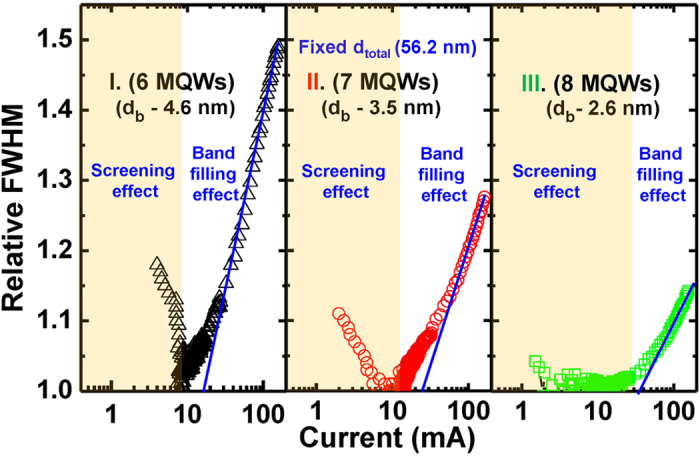
The variation of FWHM for samples I, II, and III as a function of injection current. The FWHM decreases at the low injection current region (yellow region), while it increases again with increasing injection current (white region). The former can be explained by the screening effect of internal electric field, while the latter by the band filling effect. We confirmed that the influence of the screening and band filling in QW was reduced with decreasing the barrier thickness.

Figure 5 shows a semi-log plot of full width at half maximum (FWHM), which is defined as the relative FWHM with respect to the minimum FHMW value as the injection current increases. We observed that the FWHM decreases at the low injection current region (yellow region in Fig. 5), while it increases again with increasing injection current (white region in Fig. 5). The former can be explained by the screening effect of internal electric field, while the latter by the band filling effect. Generally, as the screening effect of the internal electric field in QWs dominates, the reduction of FWHM is accompanied^24^. The decreasing trend in FWHM was appeared to reduce more obviously from sample I to sample III, implying that the internal electric field in QWs decreases as *d*_barrier_ decreases. On the other hand, the increase of FWHM is commonly known as the result of the band filling effect of the carriers in QWs at high injection carrier density region^25^. As shown in Fig. 5, the relative FWHM values measured at highest injection current (at 150 mA) decreased by about 49%, 28%, and 15% for samples I, II, and III, respectively, and the gradient of the solid lines (shown in white region) decreases as *d*_barrier_ decreases. Furthermore, the current values representing transition between screening effect and band filling effect are 8, 15 and 25 mA for samples I, II and III, respectively, showing an increase in the value as *d*_barrier_ decreases. We investigated the effective valence band potential height of the AlGaN EBL close to the *p*-GaN layer through the simulation. (see Supplementary Section IV.1) We observed that the effective valence band height was decreased with reduction of barrier thickness. Thus, we thought that the hole injection was more efficient in sample with the thin barrier due to the reduction of effective valence band height^26^. In addition, we though that the carrier density in QWs was reduced at the same injection current level with increasing *N*_W_ (and hence decreasing *d*_barrier_) due to the increased total well thickness (i.e., *d*_well_ × *N*_W_) while keeping the same *d*_active_ and *d*_well_. Furthermore, we also confirmed difference of carrier density in well through analysis of onset of high injection region in temperature-dependent *I*-*V* characteristic. (see Supplementary Section III).

**Figure 6:**
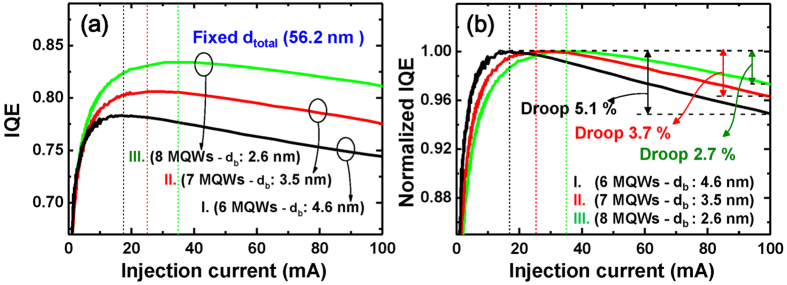
Internal quantum efficiency for samples I, II, and III as a function of injection current. **(a)** IQE and (**b)** normalized IQE versus the injection current for samples I to III. The *IQE*_*max*_ increases, and the droop decreases with decreasing the barrier thickness.

Figure 6 shows the variation of IQE and normalized IQE with increasing *I* for the three LED samples at 300 K. The IQE values were extracted from electroluminescence (EL) data by using our simplified ABC rate equation^27^, which is properly modified to fit the EL data, as will be explained below. Using this method, IQE and different contributions of radiative and non-radiative recombination processes at arbitrary injection current can be unambiguously determined without any knowledge of *A*, *B*, and *C* coefficients of the rate equation.

In the rate equation based on a well-known ABC model, total carrier generation rate (*G*) equals to the sum of Shockley-Read-Hall (SRH) non-radiative recombination rate (*An*), radiative recombination rate (*Bn*^2^), and Auger-like (and/or possibly carrier overflow) non-radiative recombination rate (*Cn*^3^), where *A*, *B*, and *C* are the respective recombination coefficients. Since integrated EL intensity (*I*_*EL*_) is proportional to radiative recombination rate, *I*_*EL*_ = *αBn*^2^, where *α* is a constant determined by the volume of the excited active region and the total EL collection efficiency. Therefore, *G* can be expressed as follows:


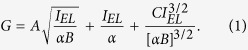


Generally, electrical carrier generation rate (*G*_electrical_) is represented as follows:


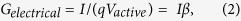


where *I*, *q*, *V*_active_, and *β*, are injection current, elementary charge, active region volume, and reciprocal of *qV*_active_, respectively. So, Eq. (1) can be expressed as follows:


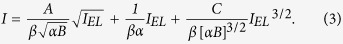


Here, 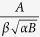, 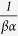, and 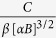 can be defined by fitting parameters *P*_1_, *P*_2_, and *P*_3_, respectively, from the injection current dependent EL experiment; thus above equation can be simplified as 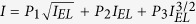. Therefore, *η* can be directly calculated from *I* as follow:


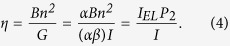


Using Eq. (4), the maximum values of IQE (*IQE*_*max*_) of samples I to III are estimated to be about 78.4% at 19.0 mA, 80.1% at 25.5 mA, and 83.4% at 35.5 mA, respectively, as shown in Fig. 5a. The efficiency droop values of samples I to III, calculated by (*IQE*_peak_ − *IQE*_100mA_)/*IQE*_peak_, are found to be 5.1%, 3.7%, and 2.7%, respectively (Fig. 6b). We found that as *d*_barrier_ decreases, both the droop-onset injection current and the overall IQE increase, while the efficiency droop becomes less severe.

**Figure 7:**
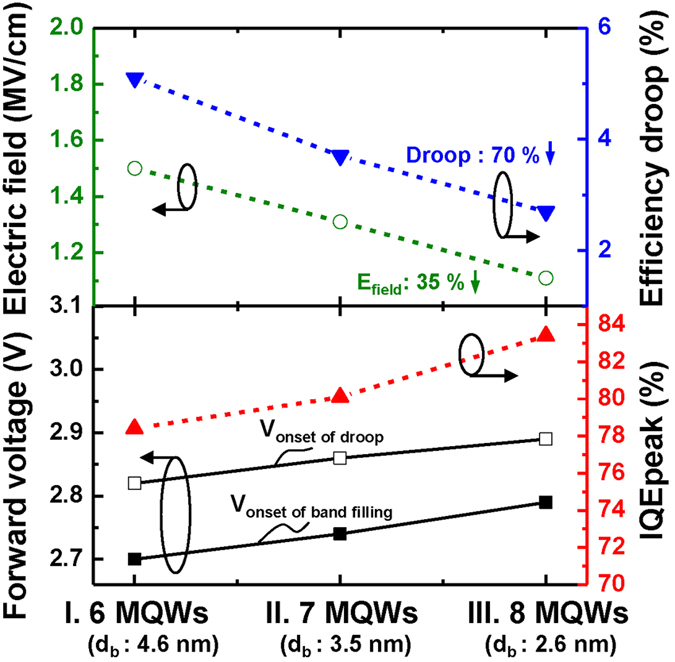
Correlation between the onset of droop and band filling, internal electric field, droop, and *IQE*_*peak*_ with varying barrier thickness. The onset voltages of droop (◻) and band filling (◾), internal electric field in well (⚪), droop (▾), and *IQE*_*peak*_ (▴) are shown for samples I, II, and III. As the barrier thickness decreases and the QW number increases, the internal electric field within QWs and droop decreases and the *IQE*_*peak*_ increases.

Figure 7 shows the onset voltages of band filling (V_onset of band filling_) and droop (V_onset of droop_), internal electric field in well, *IQE*_*peak*_, and efficiency droop. The values of V_onset of band filling_, V_onset of droop_, *IQE*_*peak*_ and efficiency droop are obtained from Figs 4 and 6 and the internal electric field in the well was calculated by only considering the influence of thickness between the well and the barrier for comparison of the three samples^26^. We found that as *N*_W_ increases and *d*_barrier_ decreases, the V_onset of band filling_, V_onset of band filling_, and IQE_peak_ increase, whereas the calculated internal electric field in wells and efficiency droop are reduced. The variation of V_onset of band filling_ and V_onset of droop_ is associated with the change in carrier density in QWs. The increase of the V_onset of band filling_ for the sample with higher *N*_W_ and thinner *d*_barrier_ indicates smaller band filling effect, which is ascribed by a decrease in carrier density in QW at the same injection condition by larger overall active volume (higher *N*_W_). The non-radiative recombination processes such as Auger or/and carrier overflow related to the high injection carrier density, are also reduced due to the reduction of the carrier density with decreasing *d*_barrier_ and increasing *N*_W_. Thus, efficiency droop is decreased for the sample with higher *N*_W_ and thinner *d*_barrier_. In addition, as *d*_barrier_ decreases, we observed that the internal electric field within QWs becomes weaken. The calculated internal electric field in wells of sample III (with highest *N*_W_ and thinnest *d*_barrier_) is decreased by 35% compared to that of sample I (with lowest *N*_W_ and thickest *d*_barrier_), which was consistent with the results of simulation and decay time (shown in Fig. 4).

## Discussion

In GaN-based LEDs, it is important to reduce the efficiency droop known as related mainly with high injection carrier density. Many efforts have been made to reduce the non-radiative recombination processes at high carrier density. To solve this problem, it is important to decrease the internal electric field as well as carrier density in QWs. The thick well, double heterostructures or simply increased QW number in the active region were used to lower the effective carrier density in QWs. In addition, the trapezoidal well, ternary or quaternary (Al)InGaN barrier were used for reducing the internal electric field in InGaN QWs. The significant reduction of internal electric field in QWs was shown in structure with same number of QWs and different barrier thickness^26^. Although these structures can reduce effective carrier density in larger active region, the material quality and recombination rate of QWs can be degraded due to the formation of additional defects and strain as well as increased quantum confined Stark effect caused by thicker active layer compared to conventional cases. (see Supplementary Section IV.2) Also, these methods for band structure engineering to decrease the internal electric field in QWs are difficult to find optimized growth conditions. Moreover, previous research for efficiency droop have merely focused on reducing either the internal electric field or the carrier density. Recently, the 3-dimensional structure such as the pyramid or the rod are applied for fabrication of LEDs with high efficiency since the carrier density and internal electric field in QWs decrease simultaneously by increase of effective active volume from the core-shell structure and utilization of semi-polar and non-polar facets. However, fabrication of LEDs using 3-dimensional structure is complicated, and many problems such as *p*-GaN growth and metal contact still remain to be solved.

Herein, we used the conventional LED structures without complicated engineering of the QW structure or post-fabrication. LED samples with a variation of quantum well number and barrier thickness are used without degradation of material quality by keeping the same active region thickness. We have systematically investigated influence of internal electric field and carrier density for efficiency droop in these LED structures. From *L*-*I*-*V* measurement, we observed that the light output power was increased in the LED sample with the thinnest barrier thickness and the largest QW number. (see Supplementary Section I.) Any material quality of the active region in samples was not observed by the variation of QW structures. In addition, the difference of internal electric field and carrier density in QWs were observed through decay time measurement by time-resolved PL, measurement of FWHM in EL spectra, and analysis of relation between the onset of high injection and the onset of efficiency droop. Furthermore, the carrier distribution in active region was investigated by using the simulation. From the IQE analysis based on our simplified ABC rate equation, it was found that overall IQE values increased, and the efficiency droop was reduced 1.7 times as the barrier thickness was reduced. From our simulation and experiment results, we observed properties such as (i) the reduction of carrier leakage by decrease of internal electric field, (ii) the improvement of hole injection due to reduction of effective valence band height, and (iii) the reduction of carrier density within QWs in sample having the thin barrier. Thus, we conclude that an enhancement of overall efficiency and a reduction of efficiency droop can be achieved by mean of strengthen of oscillator strength and reduction of non-radiative recombination processes related to high carrier density (such as Auger or/and carrier overflow) as the QW design with higher number of QWs and thinner barrier thickness is used. Although we demonstrated these improvements for blue-light emitting QWs structure in this work, this approach can be applicable for green- and ultraviolet-light emitting LEDs in the near future.

## Methods

### Experiment

The influence of droop related to the carrier density and internal electric field in QWs having different *d*_barrier_ are analyzed through various experiments. A source meter (Keithley 2400) was used for current injection. We used the integrated sphere with a fiber-coupled radiometrically calibrated spectrometer to measure the electrical and the optical properties of the LED operation under current injection. The detection of output power were performed by an array charge coupled device (Hamamatsu, S7031-1006, back-thinned CCD array). The *L-I* curve is measured in a current range of below 150 mA at room temperature. For temperature-dependent electrical experiments, the LED samples were mounted in a closed-cycle cryostat, and temperature was varied from 150 to 300 K. To carry out the time-resolved PL experiment, a mode-locked Ti:sapphire laser (Coherent, Chameleon Ultra II) was used with double frequency. The wavelength, width, and repetition rate of the pulse laser were 375 nm, 200 fs, and 200 kHz, respectively. A streak camera (Hamamatsu, C7700-01) was employed to measure the decay lifetime.

### Simulation

The simulated LED structure used for this study consists of a 0.1 μm thick GaN buffer layer, a 2.5 μm thick layer of *n*-type GaN:Si (3 × 10^19^cm^−3^), a 0.2 μm thick layer of *p*-type GaN:Mg (3 × 10^18^cm^−3^), a 0.02 μm thick layer of *p*-type Al_0.2_Ga_0.8_N:Mg (1 × 10^18^cm^−3^), and five quantum well (5QWs) structure consisting of 4.0 nm thick In_0.15_Ga_0.85_N well layer separated by 2.6, 3.5, and 4.6 nm GaN barrier layers for samples I, II, and III, respectively. The device geometry was designed with a rectangular shape of 300 μm × 300 μm. The optical and electrical properties of the LEDs are investigated numerically with the advanced physical model of semiconductor devices (APSYS)^28^, a simulation software developed by the Crosslight Software Inc. The numerical investigation is expected with a self-consistent APSYS simulation program under study, which solves Poisson’s equation, current continuity equation, carrier transport equation, and quantum mechanical wave equations. The interface charge densities caused by the spontaneous and piezoelectric polarizations are calculated automatically in simulation from the method proposed by Fiorentini *et al*.^29^. The induced sheet charge density was assumed to be 50% of the theoretical polarization due to the crystal relaxation through dislocation generation during the growth^30^. In this simulation, the Shockley-Read-Hall recombination coefficient, the radiative recombination coefficient, and Auger recombination coefficient are assumed to be 1 × 10^7^/s, 2 × 10^−11^cm^3^/s, and 2 × 10^−31^cm^6^/s, respectively. Most of the parameters used in this work are similar to those employed in literature^31^.

## Additional Information

**How to cite this article**: Yoo, Y.-S. *et al*. Effective suppression of efficiency droop in GaN-based light-emitting diodes: role of significant reduction of carrier density and built-in field. *Sci. Rep.* **6**, 34586; doi: 10.1038/srep34586 (2016).

## Supplementary Material

Supplementary Information
