# Supplementary material for: Effective suppression of efficiency droop in GaN-based light-emitting diodes: role of significant reduction of carrier density and built-in field
Source: Sci Rep. 2016 Oct 19;6:34586. doi: 10.1038/srep34586 (PMC5069459; doi:10.1038/srep34586)
Supplement: Supplementary Information [file srep34586-s1.doc]

**Effective suppression of efficiency droop in GaN-based light-emitting diodes: role of significant reduction of carrier density and built-in field**

**Yang-Seok Yoo^1^, Jong-Ho Na^2^, Sung-Jin Son^2^, and Yong-Hoon Cho^1*^**

^1^ Department of Physics, Korea Advanced Institute of Science and Technology (KAIST), 291 Daehak-ro, Yuseong-gu, Daejeon 34141, Republic of Korea

^2^ LG Innotek, LED Division, LED R&D Center, Paju 10842, Republic of Korea

*CORRESPONDING AUTHOR FOOTNOTE

Phone: +82-42-350-2549. Fax: +82-42-350-2510. E-mail: [yhc@kaist.ac.kr](mailto:yhc@kaist.ac.kr)

**I. Analysis of the electrical property for samples I, II, and III.**

The Voltage (*V*) and light output power (*L*) as a function of the current (*I*) for samples I, II, and III were measured. A source meter (Keithley 2400) was used for current injection. We used and integrated sphere with a fiber-coupled radiometrically calibrated spectrometer to measure the electrical and the optical properties of the LED operation under current injection. The detection of output power were performed by an array charge coupled device (Hamamatsu, S7031-1006, back-thinned CCD array). The *L*-*I* curve is measured in a current range of below 150 mA at room temperature. As shown in Figure S1a, the turn-on voltage and series resistance between samples were slightly different. We analyzed that increase of turn-on voltage and series resistance with reducing the barrier thickness was attributed to increase of kinetic energy of carriers traversing the QW as shown in Figure 3a. In addition, the light output power increases with decreasing barrier thickness (and hence increasing number of the wells). Figure S1b. shows that the light output power of samples II and III are about 2.7 % and 6.0 % higher than that of sample I, respectively at the maximum injection current value of 150 mA. We observed that the light output power increases with decreasing the barrier thickness without degradation of material quality in well as shown in Figure 2. We analyzed that the increase of light output power was attributed to decrease of internal electric field and increase of the radiative recombination process by improvement of the hole injection with reducing the barrier thickness.


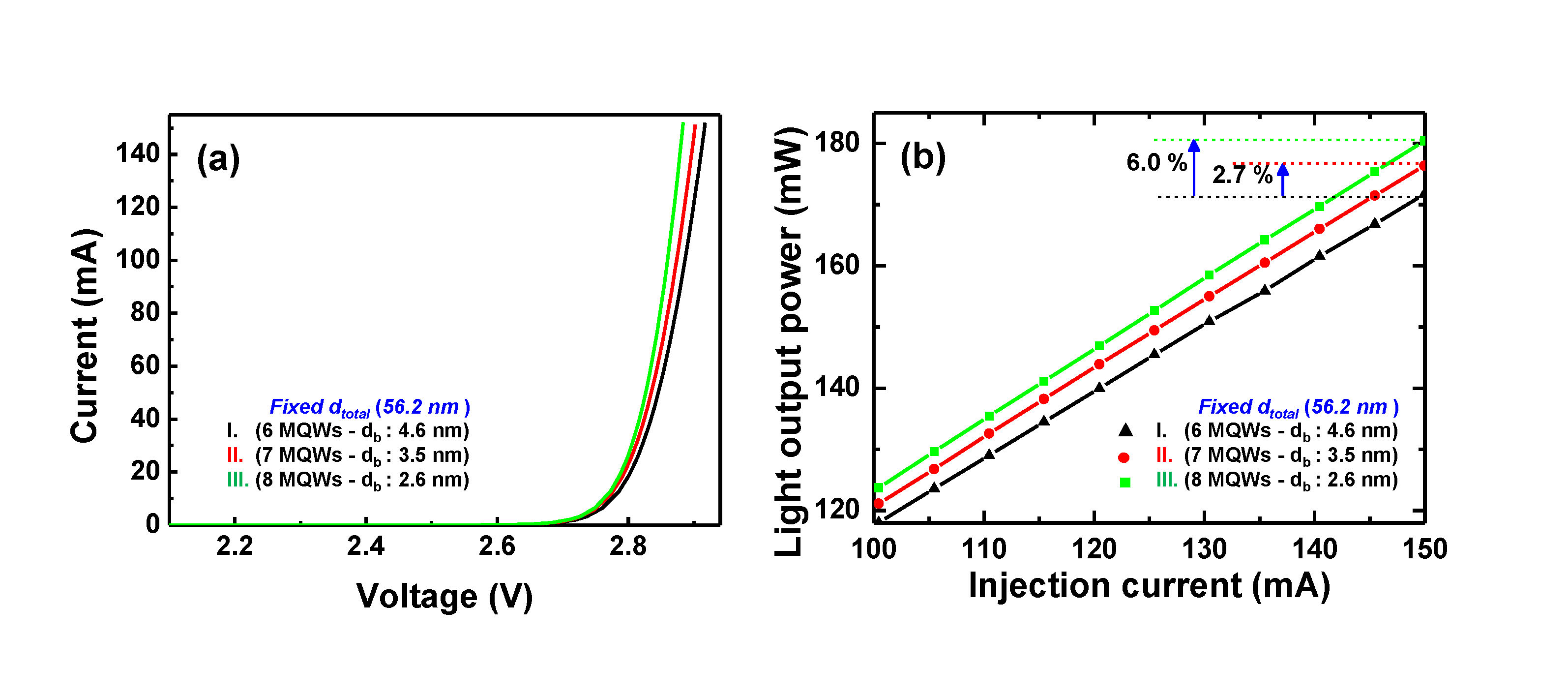


**Figure S1.** **Analysis of** **electrical property for the LED samples I, II, and III.** **a,** *I*-*V* curve, **b,** The light output power for samples I, II, and III is measured in the current injection below 150 mA.

**II. Measurement of EL spectra and peak shift for samples I, II, and III**

We measured the emission peak at the injection current 20 mA (Figure S2a), and compared the difference of peak shift between devices in the injection current range below 150 mA (Figure S2b). A source meter (Keithley 2400) was used for current injection. We used the integrated sphere with a fiber-coupled radiometrically calibrated spectrometer to measure the electrical and the optical properties of the LED operation under current injection. The detection of output power were performed by an array charge coupled device (Hamamatsu, S7031-1006, back-thinned CCD array). The *L*-*I* curve is measured in a current range of below 150 mA at room temperature. We observed that the peak wavelength between samples was different through EL spectra. First, as the barrier thickness is varied from 4.6 nm to 2.6 nm, the EL peak wavelength of LED (@20 mA) is reduced from 445.8 nm to 441.7 nm. As the LED structures were fabricated with the same growth conditions expect for the barrier growth time, we assumed that the indium composition and quantum well width were same in three samples. Thus, the shorter peak wavelength of the EL spectra for LEDs with 2.6 nm thick barrier indicated the reduced quantum confinement Stark effect (QCSE) in the active region. Second, the wavelength shift with injection current from 0 to 150 mA for LEDs with 2.6 nm thickness barrier (8 MQWs) is only 0.5 nm peak blue shifted, which is much smaller than that of LEDs with 3.5 and 4.6 nm thick (7 and 6 MQWs) barrier. The smaller blue shift of EL peak is another evidence for the reduced QCSE in the active region, indicating a reduction of the internal electric field in the active region of LEDs with decreasing barrier thickness. Although peak wavelengths were slightly different due to the difference in internal electric field between our samples, the peak wavelength difference of less than 3 nm between the samples was observed at the injection current range of 150 mA.


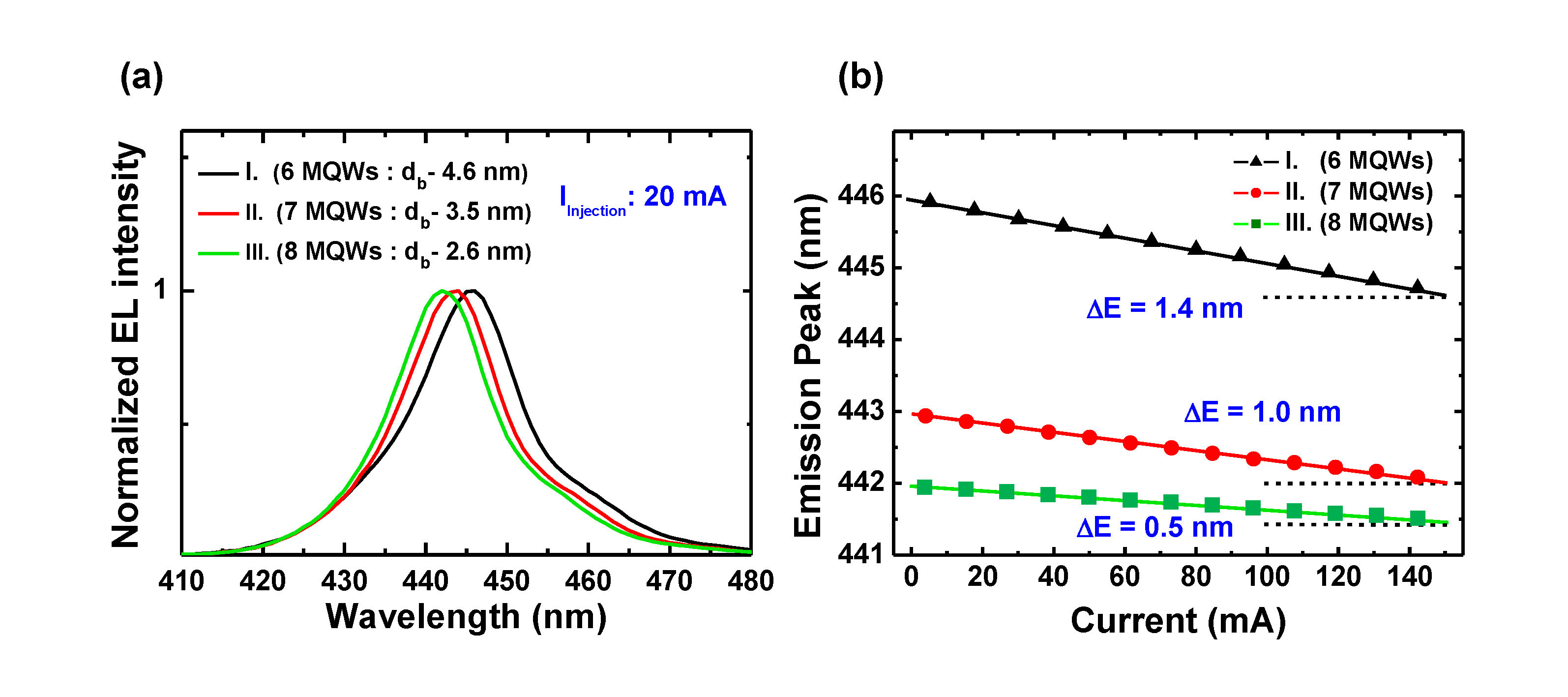


Figure S2. Comparison of peak emission and difference of peak shift for samples I, II, and III a, EL spectra at 20 mA injection current b, peak shift in a injection current range below 150 mA for samples I, II, and III.

**III. Analysis of high injection region through temperature-dependent I-V characteristics**

Recently, it was reported that correlation between the onset voltage of the high injection and the onset voltage of efficiency droop by the equation *V*_onset of high injection_ + *ΔV* ≈ *V*_onset of droop_ could exist.^1^ Figure S3 shows the correlation between the onset of high injection and the onset of the efficiency droop. The lower group of data points in Figure S3b. indicates the voltage at the onset of high injection, which is easily done by the procedure shown in Figure S3a. The onset of high injection between samples was investigated by the first derivative of the natural logarithm injection current (*I*) with respect to the diode voltage (V), i.e., (dLn (*I*) / d*V*). Figure S3a. is an example of the result related to the onset of high injection for sample I at different temperature, which displays clear separation between the low-injection and the high injection region and shows decrease of forward voltage indicated demarcation between the two injections as temperature increases. In addition, the upper groups of data points in Figure S3b. indicate that the voltage is related to the peak values of internal quantum efficiency (IQE) as a function of temperature. The relation between two groups shows that the onsets of high injection and efficiency droop decrease together with a constant voltage difference, *ΔV,* as the temperature increases. We observed that the *ΔV* was determined to be about 0.3 V in samples I to III. Generally, the *ΔV* in high injection region is related to the carrier mobility, which is affected by the impurity concentration such as donor and acceptor concentration or electron and hole concentration. Since other growth conditions of samples I to III with the exception of barrier thickness are same, we believe that the *ΔV* in samples I to III is identical. In addition, we observed that the onset of the high injection voltage (or onset of droop voltage) becomes smaller as the barrier thickness increases. The relatively large carrier density in the sample with thick barrier leads to an earlier onset of high injection level compared to the sample with thin barrier. Thus, the non-radiative recombination related to the high injection carrier density such as the carrier overflow or leakage can occur more easily in the sample with thicker barrier, and consequently the largest efficiency droop is observed in sample I as shown in Figure 5.


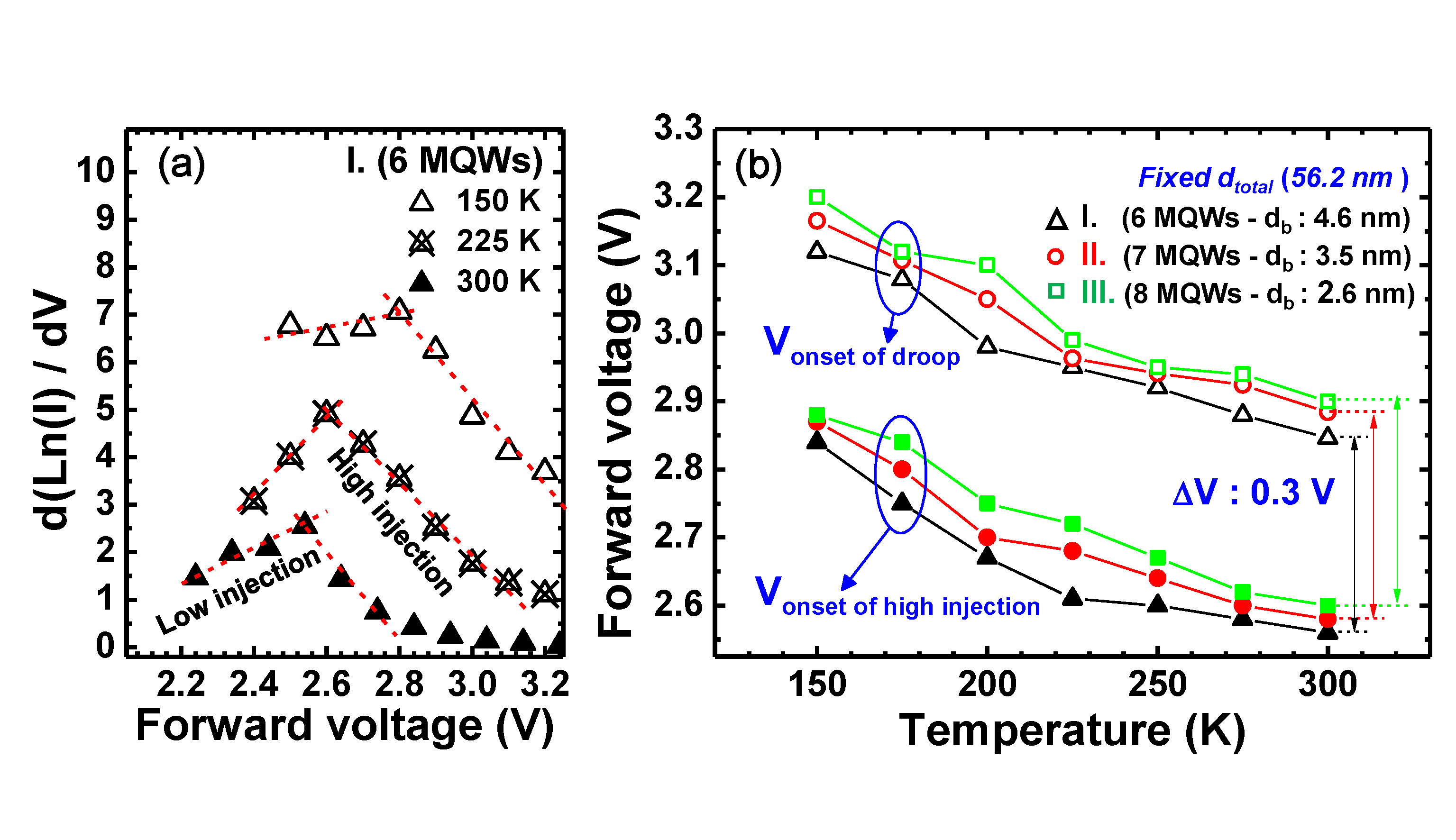


Figure S3. Correlation between the onset of high injection and onset of droop a, Determination of the onset of high level injection point as the value of voltage where the slopes of low and high injection cross each other with increasing voltage. b, Voltage at the onset of high injection and voltage at the onset of the efficiency droop (i.e., voltage at the maximum efficiency) as a function of temperature.

**IV. Simulation analysis of for different sample structures**

**IV.1) Comparison of the case with the same number of QWs and different barrier thickness**

We compared our case with the case of the same number of QWs and different barrier thickness through simulation. Figure S4.1a shows the schematic of structures used in the simulation. Figure S4.1b indicates the valence band diagram (solid line) and quasi-Fermi level (dash line), and we investigated the effective valence band barrier height of the GaN barrier close to the p-GaN, which was defined as (△Φ) in Figure S4.1b below. We found that the △Φ for samples with increased barrier thickness compare to our structures used in this study was much larger. The hole distribution for samples used in this study was much improved than that of the samples having increased barrier thickness as shown in Figure S4.1c. From these simulation results, we thought that the utilization of thin barrier was very helpful for hole injection. In addition, it is reported that the internal electric field of QWs is reduced in the sample with thin barrier.^2^ Thus, we can expect the reduction of carrier leakage in the sample with the thin barrier thickness. The explanation for the improvement of hole injection and the reduction of internal electric field in the QW was written in Figure 3 of manuscript.


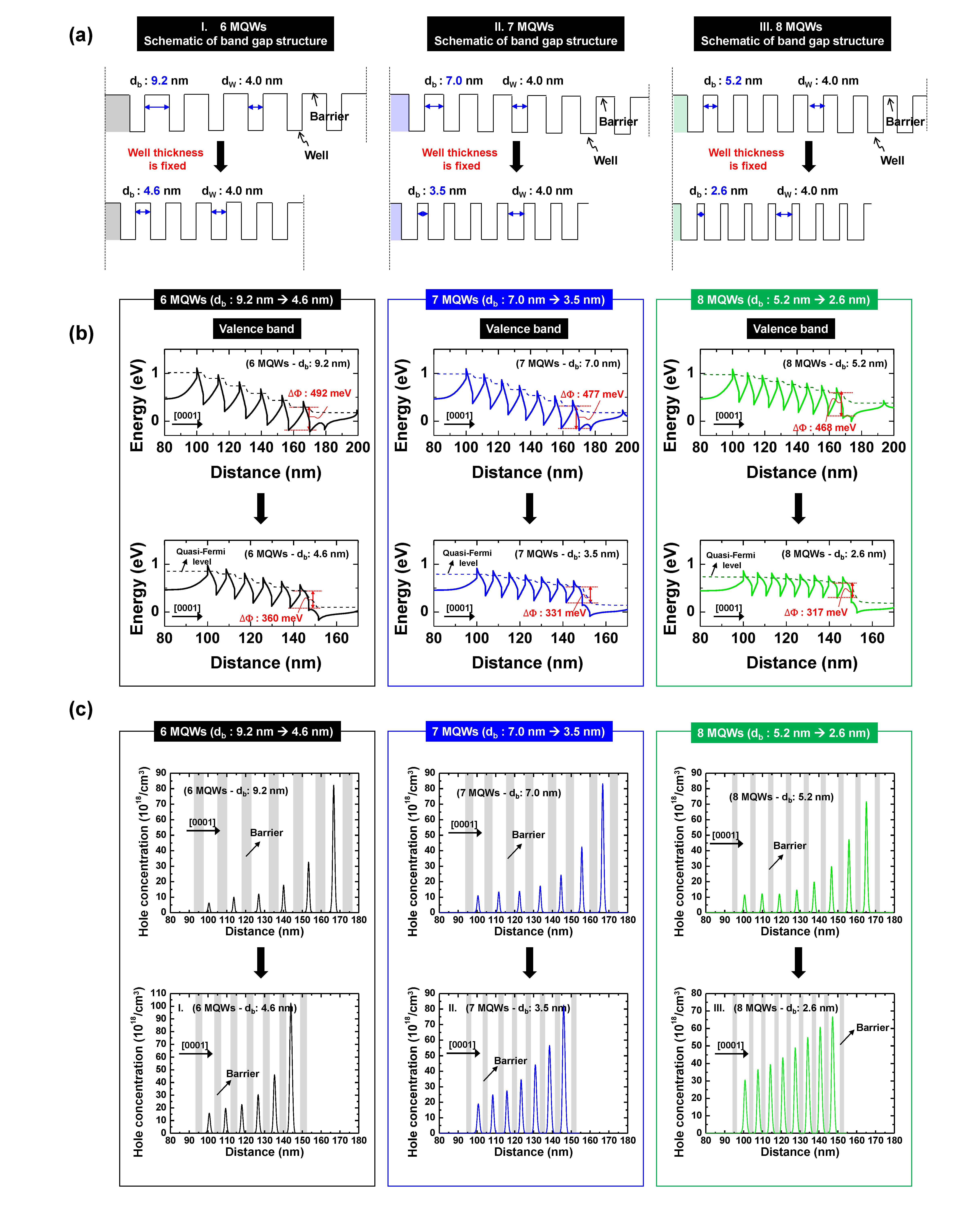


Figure S4.1 Simulation results for samples I, II, and III. a, schematic of structures used in simulation b, valence band diagram c, hole concentration at 50 A/cm^2^.

**IV.2) Comparison of the case with the different number of QWs**

By using structures with a large number of QWs or thick QWs, we can suppress the efficiency droop due to reduction of carrier density in QWs by increase of active volume. However, the device performance can be limited by the degradation of crystal quality with increasing the QW number and the induced strain by increased the well thickness.^3^ Recently, it was reported that the efficiency droop was decreased by using the sample with thin barrier (fixed number of QWs) due to reduction of internal electric field in QWs. We investigated the difference of hole distribution through the simulation for samples a, b, and c. The schematic of conduction band for structures used in simulation was shown in Figure A below. The samples a and b shown in the schematic are same with structures I and III used in this study. The well and barrier thickness for samples a and b are same, while the number of QWs is different. The valence band diagram and quasi-Fermi level are shown in Figure B below. The effective barrier height for the barrier close to p-GaN layer is defined as △Φ. We found that the simply increase of QWs number could not help for hole injection, and the hole distribution was still concentrated in the QW close to the p-GaN layer (as shown in the result between samples a and b of Figure C below) However, significant hole injection improvement was observed in samples c compare to the samples a and b. We thought that the improvement of hole distribution for sample c was attributed to reduction of carrier density by increase of the QW number, reduction of effective barrier height, and decrease of carrier leakage by small internal electric field in active region.


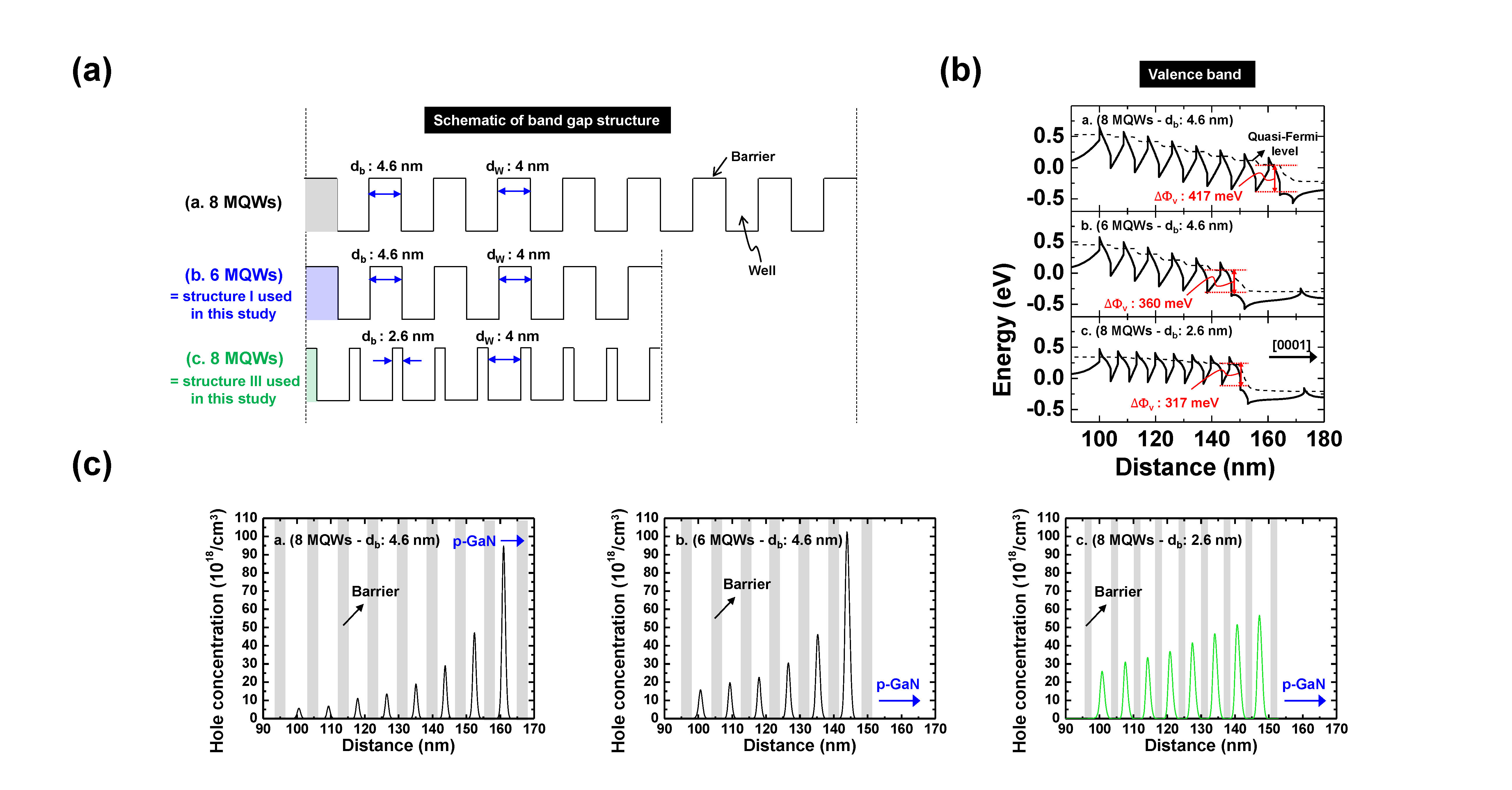


Figure S4.2 Simulation results for samples a, b, and c. a, schematic of structures used in simulation b, valence band diagram (solid line) and quasi-Fermi level (dash line) c, hole concentration at 50 A/cm^2^.

**Reference**

1. Meyaard, D. S. *et* *al*. Identifying the cause of the efficiency droop in InGaN light-emitting diodes by correlating the onset of high injection with the onset of the efficiency droop. *Appl. Phys. Lett.* 102, 251114 (2013).
2. Ni, X., Fan, Q., Shimada, R., Özgür, Ü. and Morkoç, H. Reduction of efficiency droop in InGaN light emitting diode by coupled quantum wells. *Appl. Phys. Lett.* 93, 171113 (2008).
3. Zhang, Y. P. *et* *al*. Nonradiative recombination – critical in choosing quantum well number for InGaN/GaN light-emitting diodes. *Opt*. *Exp*. 23, A34 (2015).
